# Mental Health and Health-Related Quality of Life in Austrian Adolescents with Chronic Physical Health Conditions: Results from the MHAT Study

**DOI:** 10.3390/jcm12051927

**Published:** 2023-02-28

**Authors:** Gudrun Wagner, Andreas Karwautz, Julia Philipp, Stefanie Truttmann, Wolfgang Dür, Karin Waldherr, Gabriele Berger, Michael Zeiler

**Affiliations:** 1 Department for Child and Adolescent Psychiatry, Medical University of Vienna, 1090 Vienna, Austria; 2 Department of Sociology, University of Vienna, 1090 Vienna, Austria; 3 Department for Research and Development, Ferdinand Porsche FernFH—Distance Learning University of Applied Sciences, 2700 Wr. Neustadt, Austria; 4 Department of Pediatrics and Adolescent Medicine, Medical University of Vienna, 1090 Vienna, Austria; 5 Pediatric Diabetes Outpatient Clinic, OEGK Health Care Centre Vienna Floridsdorf, 1210 Vienna, Austria

**Keywords:** mental health, quality of life, adolescents, epidemiology, chronic physical health conditions

## Abstract

Chronic physical health conditions (CPHC) are on the rise in younger age groups and might have a negative impact on children and adolescents. In a representative sample of Austrian adolescents aged 10–18 years, internalizing, externalizing, and behavioral problems were assessed cross-sectionally using the Youth Self-Report and health-related quality of life (HrQoL) using the KIDSCREEN questionnaire. Sociodemographic variables, life events, and chronic illness specific parameters were considered as associated variables with mental health problems in individuals with CPHC. Of 3469 adolescents, 9.4% of girls and 7.1% of boys suffered from a chronic pediatric illness. Of these individuals, 31.7% and 11.9% had clinically relevant levels of internalizing and externalizing mental health problems, respectively, compared to 16.3% and 7.1% adolescents without a CPHC. Anxiety, depression, and social problems were twice as high in this population. Medication intake due to CPHC and any traumatic life-event were related to mental health problems. All HrQoL domains were deteriorated in adolescents with a double burden of mental and CPHC, whereas adolescents with a CPHC without mental health problems did not differ significantly from adolescents without a chronic illness. Targeted prevention programs for adolescents with a CPHC are urgently needed to prevent mental health problems in the long term.

## 1. Introduction

Chronic physical health conditions (CPHC) are often associated with diverse physical, psychological, and social functional limitations, leading to a burden of illness over a sustained period of time [1]. Using a generic or non-categorical definition, chronic health conditions are defined as disorders having a biological, psychological, or cognitive basis, last for at least one year, and produce one of the following sequelae: (a) limited function, activities, or social roles within physical, cognitive, emotional, and social development; (b) connection with a reliance on medication, special diet, medical devices, or personal assistance; (c) the need for medical care or related psychological or educational services [2,3]. In the present paper, we refer to chronic physical conditions only.

Epidemiological studies show a range of between 10% and 31% of children and adolescents who are affected by CPHC, depending on the age range and specific diseases included [2,4], with a tendency for increasing incidence of several conditions such as diabetes and cancer [2].

In the Austrian Health Behavior in School-aged Children (HBSC) survey of 2018, 19% of adolescents showed a chronic condition, which is an increase compared to 2010 (16%) and 2006 (14%). An increase by age, especially in girls, has been observed in all three survey waves. In girls, 16.6% of 11-year-old adolescents reported a CPHC, while this percentage increased to 24.1% in 17-year-olds. In boys, the prevalence increased from 13.5% in 11-year-old adolescents to 20.2% in 17-year-olds [5,6,7]. These are lower rates compared to data from the United States, where 31% of all US children suffer from a CPHC [8].

Although many people adjust well to a chronic condition, estimates of a psychological comorbidity in this population amount to 20%, which is about double compared to healthy young people [9,10,11]. Further population-based studies and reviews confirmed that people with CPHC are at an increased risk for behavioral and mental health problems [4,12,13]. However, there is a relative dearth of focus on the impact of CPHC on mental health in children and adolescents [2,14]. Cross-sectional associations of CPHC with a preponderance of internalizing disorders, especially anxiety and depression, compared to externalizing disorders have been previously reported, while few longitudinal population-based studies [4,13,15,16] and meta-analyses [14,17] exist. Early age at onset predicted externalizing problems [16]; other surveys state that illness-specificity or gender is associated with elevations in externalizing problems [17]. Overall, small sample sizes, a focus on clinical settings, and a lack of adolescent self-reports have been criticized in previous studies [16]. Existing meta-analyses highlight the need for a specific focus on younger populations [14], while a differentiation between childhood and adolescence is necessary, as it is assumed that poor mental health in this vulnerable population might be associated with CPHC, and a chronic disease might disrupt normal developmental milestones of this period, such as increased autonomy from parents [18].

Factors that contribute to the elevated risk of mental health problems in this population are manifold and may include biological, psychological, and social aspects. Common stressors in CPHC such as treatment regimens and medical monitoring place significant demands on families, increase stress not only in the child but also among parents, and may lead to overdependence in the parent–child relationship [19]. These illness-related demands may prevent children from engaging in social activities that would strengthen friendships and self-development. Illness-related demands may even have a more detrimental impact during adolescent years. The adolescent period is viewed as a critical period of social development and identity formation. Existing evidence suggests that bio-psychosocial changes in adolescence (including pubertal hormones associated with changes in the reward and stress systems) may have a substantial impact on the adjustment of adolescents to their CPHC. They may see the demands and restrictions imposed by their treatment regimen as a barrier to this normative experimentation and separation from parents [1]. They are at risk of delayed attainment of developmental milestones compared to their peers [20]. Trying to appear as “normal” is a major focus of adolescents with CPHC [21], potentially leading to more negative illness cognitions, especially in conditions with physical manifestations of the illness (i.e., insulin pumps in diabetes). Such aspects may put these adolescents more at risk of experiencing victimization during a period of heightened social development. Increased levels of peer victimization have consistently been reported [22]. Contemporary theories view CPHC as a stressor in early development and as an additional barrier to self-esteem and social development in adolescence [2]. Furthermore, mental illness is viewed as an outcome of predisposing and precipitating stressors; however, evidence from epidemiological studies is contradictory [15].

Mental health issues are often neglected in medical care. Adolescents with CPHC are often not systematically screened for mental health problems, and referral to mental health care often fails [23,24]. Indeed, the Lancet series on Global Mental Health concluded that mental health should be incorporated into all aspects of health [2]. Thus, analyzing the prevalence of behavioral problems and mental health risks of children and adolescents with a CPHC helps to understand psychosocial consequences and provides information on who should be screened for mental health problems and the need for prevention in this population [17]. Moreover, an in-depth investigation of which sociodemographic and disease-specific factors contribute to an increased risk for mental health problems in children and adolescents with CPHC is of high importance for the planning of targeted prevention efforts. 

Although associated with mental health, the concept of health-related quality of life (HrQoL) can also be regarded as a highly relevant outcome variable for individuals with CPHC. HrQoL is seen as a “multidimensional construct covering physical, emotional, mental, social, and behavioral components of well-being” (p. 295) and thus provides important information about how an individual copes with everyday life [25]. All the aforementioned aspects discussed in relation with an elevated risk for mental health problems in young people with CPHC (including self-perception, parent-child relation, social relations with peers, and school environment) are also considered in current HrQoL concepts [26]. Indeed, the existing research suggests that HrQoL and psychosocial functioning might be impaired in adolescents with CPHC [27]. However, there is also evidence that some affected adolescents show an impaired HrQoL while others do not, depending on various factors, such as resilience, personality factors, and family support [28]. Maintaining a good quality of life despite the adversities associated with CPHC may be one of the key goals in this population. Thus, investigating the question of who can maintain a high HrQoL and which factors contribute to an impaired HrQoL is highly relevant, also with regard to the development of targeted prevention initiatives. 

The Mental Health in Austrian Teenagers (MHAT) study is the first large epidemiological study collecting data on mental health in a representative national sample of adolescents aged 10–18 years in Austria. This study also aims to assess the prevalence of CPHC in the adolescent population, determining comorbid mental health problems in adolescents with and without chronic conditions and associated HrQoL. Moreover, risk factors for developing mental health problems in adolescents with chronic conditions should be identified. 

Therefore, the aims of our study are (1) to test whether adolescents with CPHC obtained from a representative population survey have higher levels of internalizing and externalizing mental health problems compared to adolescents without CPHC; (2) to assess correlates with elevated mental health risks in the population of adolescents with CPHC including sociodemographic characteristics, disease specific characteristics, chronic diseases in the family, and stress-full life events, and (3) to assess whether a CPHC per se or in combination with mental health problems has impact on several HrQoL domains, including self-perception, family relations, peer-group relations, school performance, and social acceptance. At the European level, the current study is one of the largest population-based surveys on this topic and thus provides valuable insights into the impairment in mental health and HrQoL as well as associated factors that may further guide the development of preventive efforts for children and adolescents with CPHC.

## 2. Materials and Methods

### 2.1. Participants and Recruitment

In this study, we used data from the ‘Mental Health in Austrian Teenagers’ (MHAT)-study, an epidemiological survey that aimed to obtain the prevalence of mental health problems in a large representative sample of Austrian adolescents aged 10 to 18 years. The sample was recruited via schools including all school types in all regions of Austria. First, all secondary schools in Austria were informed about the study and asked to participate. Of the schools willing to participate (n = 261), school classes of the 5th, 7th, 9th, and 11th grade were randomly selected for this study (maximum of 2 classes per school). The selection of classes was stratified by school type and region, resulting in a selection representative for the Austrian landscape of schools. Subsequently, all students within the selected classes were invited to participate, informed about the procedure, and asked to provide informed consent. Inclusion criteria included (1) to be student of a selected class, (2) provision of written informed consent, and (3) to have sufficient German language skills to understand the questionnaire items. Written informed consent was collected from all participants and legal representatives prior to the inclusion in the study. Once the informed consent forms were collected, the participants completed a questionnaire battery to assess the relevant outcome variables (see below). The questionnaire was either completed in a paper-and-pencil or online format, based on the technical equipment of schools, during a school lesson of 50 min. Equivalence between the paper-and-pencil and online questionnaire formats was confirmed [29]. The whole procedure was moderated by a class teacher who received detailed instructions on how to moderate the assessment. The feasibility of this procedure for the teachers was previously checked in a pilot study [30]. 

Finally, a total of 3610 adolescents from the school sample participated in this study. The response rate was 47.3%; the most common reason for non-response was failure to bring signed parental consent to school on the day of data collection. A total of 129 individuals did not provide data on chronic diseases; thus, a sample of 3481 was finally included in the analysis. 

Ethical approval was obtained from the Ethics Committee of the Medical University of Vienna (#1134/2013). More details about the sampling, recruitment strategy, and procedures are published in Zeiler et al. [31] and Wagner et al. [32], both of which are open-access.

### 2.2. Measures

#### 2.2.1. Sociodemographic Information and Chronic Physical Health Conditions (CPHC)

Apart from sociodemographic information (e.g., sex, age, migration background, socioeconomic status assessed with the Family Affluence Scale [33], living situation, diagnosed somatic and psychiatric disorders in the family) that were used to describe the sample, data on CPHC, mental health problems, and quality of life were included. CPHC were self-reported by the adolescents as a yes/no answer to the question, “Do you suffer from a physical illness or handicap diagnosed by a medical doctor?” The following categories were specified and checked when applicable: diabetes, hypertonia, arthritis, paralysis, asthma/chronic obstructive bronchitis, epilepsy, migraine/headache, orthopedic disease, thyroid disease, cancer, heart disease, gastric disease, allergy, physical handicap, or others (with the possibility of specification). We did not include mental health issues or obesity. Furthermore, illness onset (age of illness onset in years), as well as the necessity of regular medication intake due to the CPHC (yes/no) and regular medical visits (yes/no), were assessed. Allergies were rated as CPHC only when regular medication intake or regular medical visits were necessary. Chronic health conditions (both physical and mental) of other family members (parents, sisters) were collected in the same manner. Additionally, adolescents rated their school performance on a 4-point scale (very good to below average).

#### 2.2.2. Mental Health Problems

The Youth Self-Report (YSR: [34], German version: [35]), a widely used mental health screening questionnaire [36] to assess emotional and behavioral problems, was used to obtain the prevalence of adolescents at risk for mental health problems. The 103 problem items, measuring behavioral and emotional problems on a three-point scale (0 = not true, 1 = somewhat or sometimes true, 2 = very true or often true) over a six-month time period, are summed into eight syndrome scales (withdrawn, somatic complaints, anxious/depressed, social problems, thought problems, attention problems, delinquent behavior, and aggressive behavior) and three broadband scales (total problem score, internalizing problems, externalizing problems). For the broadband scales, good internal consistencies are reported (Cronbach’s alpha > 0.86); for the syndrome scales, Cronbach’s alpha is 0.56–0.86. YSR ratings are summed per scale and raw-scores are transferred into T-scores using German norm data. Higher scores indicate higher levels of mental health problems. As described in the manual, cut-off scores of *T* > 63 for the broadband scales and *T* > 69 for the syndrome scales are used to define clinically relevant high-risk cases.

#### 2.2.3. Health-Related Quality of Life (HrQoL)

The KIDSCREEN questionnaire [37] was used to obtain several domains of HrQoL, including a global measure of HrQoL (Kidscreen-10), self-perception, parent relations and home life, social support and peers, school environment, and bullying (i.e., social acceptance). This measure can serve as a proxy for psychosocial impairment in different domains. Items are rated on a 5-point Likert scale. Internal consistencies were good for all used dimensions (Cronbach alphas of 0.77–0.89). Raw scores were transferred into T-Scores according to the available German normative data. 

### 2.3. Data Analyses

The statistical analysis was performed with IBM SPSS Statistics 27.0 (IBM Corporation, Armonk, NY, USA, 2020) A global significance level of α = 0.05 was set for the statistical tests. First, the prevalence of CPHC and several groups of CPHC was calculated for the entire sample. Inverse probability weighting by gender and age group was used to adjust for deviations from the sampling plan, which reflected the Austrian general population of adolescents. Additionally, prevalence figures were calculated separately for gender. Next, we compared sociodemographic characteristics between adolescents with vs. without a CPHC using chi² tests. Furthermore, we analyzed whether the group of adolescents with a CPHC differ from those without a CPHC regarding the percentage of clinically relevant mental health problems (YSR total and syndrome scales) using chi² tests and regarding YSR scores using *t*-tests. To account for multiple testing, the significance level of tests for differences in the YSR syndrome scales was Bonferroni-corrected (8-syndrome scale, adjusted α = 0.006). Moreover, we conducted univariate logistic regressions to predict clinically relevant mental health problems in the sample of adolescents with a CPHC. In this analysis, clinically relevant mental health problems were defined as scoring above the clinical cut-off in at least one of the YSR broadband or syndrome scales. We considered a wide range of sociodemographic variables (gender, age group, family status, migration background, socioeconomic status, place of residence, parental employment status), potentially stressful life events (chronic or psychiatric disorders in the family, any burdensome of traumatic life event), and disease-specific variables (age of onset, number of CPHCs, necessity of regular medical checks or medication intake due to the chronic disorder) as potential predictors. Potential predictors with *p* < 0.10 in the univariate analyses were further included in a multivariate logistic regression model. Finally, we conducted general linear models to analyze group differences in HrQoL scores between adolescents with a CPHC and a comorbid mental health problem, adolescents with a CPHC but without a mental health problem, and healthy individuals without a CPHC or mental health problem. Group differences in HrQoL domains were tested on a Bonferroni-adjusted significance level of α = 0.008.

## 3. Results

### 3.1. Prevalence of Chronic Physical Health Conditions

Overall, 8.3% [95%CI: 7.4; 9.2] of the adolescents reported any CPHC, with a slight preponderance of girls (9.4%, [95%CI: 8.1%; 10.7%]) compared to boys (7.1%, [95%CI: 5.8%, 8.44]). The highest rates were assessed for orthopedic problems (1.9%) and asthma (1.9%), followed by allergies (1.8%) and headaches including migraine (1.4%). For orthopedic problems and migraine, the prevalence for girls was about twice as high than for boys. For all other conditions, the prevalence was below 1% (Table 1). A percentage of 22.5% suffered from more than one CPHC; 33.7% of those with a CPHC reported regular medication intake and 39.7% the need for regular medical visits. The mean age of onset was 7.05 years (SD = 5.61; range 0–12), and the mean duration of illness was 8.23 years (SD = 5.37; range 3–12).

Older children were more often affected than younger children (*p* < 0.001). Moreover, adolescents with a CPHC reported a higher percentage of having a parent or sibling also suffering from a CPHC (43.4% vs. 12.6%, *p* < 0.001) or psychiatric disorder (7.3% vs. 4.0%, *p* < 0.001) as well as deteriorated school performance (*p* = 0.034, Table 2). No differences were reported for SES, migration background, residency, family status, or parental employment (Table 2).

### 3.2. Prevalence of Mental Health Risk in Adolescents with and without Chronic Physical Health Conditions

**Figure 1:**
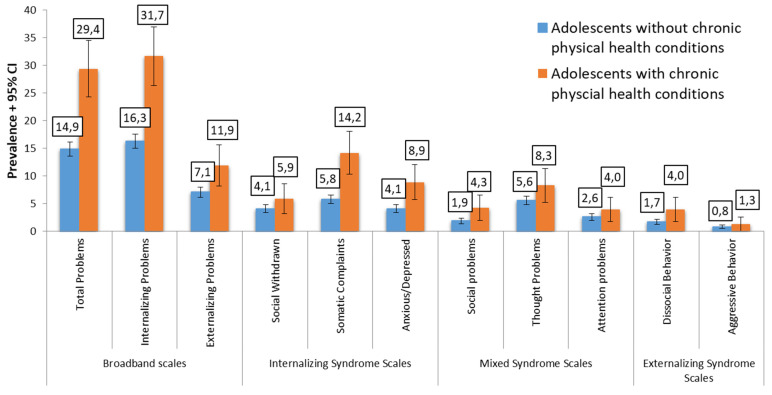
Prevalence (%) of clinically relevant mental health problems in adolescents with vs. without chronic physical health conditions.

Clinically relevant YSR total problem scores and internalizing problems were almost twice as high in the group with CPHC (29.4% and 31.7%) compared to adolescents without a CPHC (14.9% and 16.3%; both *p*-values < 0.001). Clinically relevant externalizing problems were also significantly more prevalent in adolescents with a CPHC (11.9% vs. 7.1%; *p* = 0.003). Regarding YSR syndrome scales, the prevalence of somatic complaints was almost threefold (14.2% vs. 5.8%, *p* < 0.001), and anxiety and depression symptoms were about twice as high in adolescents with a CPHC (8.9% vs. 4.1%; *p* = 0.001). Clinically relevant social problems were about twice as prevalent in the group with a CPHC (4.3% compared to the adolescents without CPHC (1.9%, *p* = 0.006)). Social withdrawal, thought problems, attention problems, dissocial behavior, and aggressive behavior did not significantly differ between groups (see Figure 1). When analyzing YSR scores, adolescents with CPHC had significantly higher levels of mental health problems in all domains (all *p*-values < 0.001), while the effect sizes were in the low-to-medium range (see Supplementary Table S1 for details.)

### 3.3. Associated Variables with Mental Health Problems in Chronic Physical Health Conditions

We aimed to identify psychosocial variables associated with mental health problems in the group of adolescents with CPHC. Univariate analyses identified psychiatric disorders of a parent or sibling (*p* = 0.050), any traumatic life event (*p* = 0.001), and regular medication intake due to the CPHC (*p* = 0.001) as associated factors for mental health problems in adolescents with a CPHC (Table S2). Moreover, the number of CPHCs tended to be associated with mental health problems (*p* = 0.089) and was therefore included in the multivariate model. In the multivariate model, any traumatic life event (*p* = 0.002) and the need for regular medication intake (*p* = 0.001) significantly predicted clinically relevant mental health problems in adolescents with a CPHC (Table 3). The omnibus model test yielded a significant result (chi² (5) = 31.489, *p* < 0.001; Cox–Snell R² = 0.104, Nagelkerke R^2^ = 0.143).

### 3.4. Health Related Quality of Life in Adolescents with Chronic Physical Health Conditions and Mental Health Problems vs. without Mental Health Problems and Healthy Controls

Adolescents with a CPHC and comorbid clinically relevant mental health problems had by far the lowest HrQoL scores in all domains and strongly differed from chronically ill adolescents without mental health problems and healthy adolescents (Table 4). Regarding overall HrQoL, as well as the self-perception and school environment domains, adolescents with a CPHC but without mental health problems had significantly lower scores compared to healthy adolescents, whereby mean differences were low. Regarding the parent relations and home life, social support and peers, and bullying subscales, chronically ill adolescents without mental health problems did not differ from healthy adolescents.

## 4. Discussion

In our representative sample of adolescents in Austria, we found that clinically relevant mental health problems were almost twice as high in adolescents with chronic conditions (29% vs. 15%), especially for internalizing syndromes such as anxiety and depression (9% vs. 4%) and social problems (4% vs. 2%). Potentially traumatizing life events and regular medication intake were related to mental health problems in this population.

This finding is in line with the results of systematic reviews confirming higher prevalence rates of anxiety disorders in youths with CPHC compared to the general population [38]. Associations between mental disorders and CPHC were reported in 35% of adolescents in a representative cohort in the US [39]. The combination of physical disorders and anxiety disorders occurred in 21% of the population. Moreover, some evidence for the association between anxiety and adverse disease-related outcomes has been revealed for some of the CPHCs (asthma and inflammatory bowel disease), whereas for others, anxiety was associated with worse as well as better treatment adherence (diabetes) [38]. However, in diabetes, internalizing disorders were only elevated in adolescents who were manipulating their insulin dose, whereas rates of psychiatric comorbidity in adolescents without management problems were comparable to adolescents without a chronic illness [40]. The experience of anxiety is associated with poor prognosis if untreated, as well as with the development of other mental health problems and psychosocial impairments such as deteriorated academic achievement and peer relationships [38].

Higher levels of depression have been found in adolescents with CPHC, with larger effect sizes in studies with a higher proportion of girls [17,41]. Reasons for elevated depression rates have been attributed to the burden of the CPHC (such as symptom exacerbation, daily care regimen) that in turn can affect social relationships, which are crucial for positive development, especially in adolescence [21]. Long-term outcome studies have shown that this comorbidity on the one hand influenced treatment outcome of the illness (such as decreased metabolic control, treatment adherence increased hospitalization in diabetes), and on the other hand affected quality of life, disability, and pain from other diseases (such as heart disease, arthritis) [42]. In adults, comorbidity of a CPHC with depression increased functional disability and absenteeism from work independent from the illness type [43]. Our results add to these findings and show that having a CPHC can lead to a deteriorated school performance, which might have effects on work performance in adulthood.

The co-occurrence of mental and physical conditions could have synergistic effects on disability through underlying pathophysiology associated with the functioning of the autonomous nervous system (sympathetic–adrenal–medullary system) and the neuroendocrine system (hypothalamic–pituitary–adrenocortical or HPA axis). Disturbances in both systems have been associated with anxiety and depression and with a range of physical disorders mediated by the cumulative burden of chronic somatic and mental health disease [44].

Other mechanisms include the possibility that depression may exacerbate the disabling effect of a CPHC through its influence on treatment adherence and health behaviors. Moreover, depression may interfere with adjustment to physical conditions. Finally, it is possible that mental comorbidity is a marker of physical condition severity [44].

In summary, there seems to be a vicious cycle that starts with the burden of illness, potentially leading to mental health problems, which in turn has a negative effect on treatment outcome and deteriorates the CPHC, leading to an even higher burden of illness that is associated with worse quality of life and school/work performance. Therefore, it is crucial to support adolescents with CPHC from the time of diagnosis with psychological interventions. Illness acceptance and coping with the CPHC and prevention of mental health problems can be fostered. For those already suffering from a co-morbidity of physical and mental condition, psychotherapeutic interventions should be provided to disrupt this vicious circle. A proactive discussion about mental health at the pediatric hospital in charge of the child and reference to appropriate mental health care services are preferred by parents of children with CPHC [45]. The application of motivational interviewing techniques might be useful to foster psychotherapy uptake [24]. Especially for adolescents, Internet or mobile-based interventions might also be feasible [46].

There is evidence that the COVID-19 pandemic may have further increased mental health problems (particularly depression, anxiety, and stress symptoms) in children and adolescents with a CPHC [47,48], which underlies the current importance of mental health care interventions in this population.

In our study, regular medication intake and the presence of a potentially traumatizing life event were related to mental health problems with a more than twofold and threefold increase in risk, respectively. Traumatic life events covered physical and sexual abuse within the family and bullying at school.

It is a well-known fact that childhood trauma, especially during key periods of CNS development and maturation, increase the vulnerability for developing mental health problems such as depression, anxiety, post-traumatic stress disorder, substance use disorders, personality disorders, and schizophrenia. The interaction between biological and psychosocial risk factors increase the risk for development of psychiatric disorders [49]. It was previously shown in an adult sample that the history of interpersonal trauma exposure is associated with anxiety symptoms, depressive symptoms, increased alcohol use (i.e., frequency and quantity), and trauma-related distress [50]. Furthermore, anxiety and mood disorders tend to persist into adulthood if untreated [51]. Hence, low-level access to therapeutic and psychological interventions for trauma is needed in order to prevent additional mental health problems in this vulnerable population [52]. The need for regular medication intake may be an indicator for the severity of CPHC, elevating the burden of disease and therefore the risk for mental health problems and psychological distress [53].

One of our key findings is that HrQoL in adolescents with a physical condition but without a comorbid mental health problem only marginally differed from healthy adolescents. However, having an additional mental health problem in chronically ill patients was associated with distinct deterioration of HrQoL in all domains, including self-perception, parent relation, peer relation, school environment, and bullying (i.e., social acceptance).

Self-perception is part of the multidimensional construct of the self-concept that comprises global evaluations of the self, such as self-esteem and domain-specific perceptions such as competence and appearance. Results of a meta-analysis have been inconsistent, showing both lower and equal self-esteem in children and adolescents with and without chronic diseases [54]. Our results add to these findings by showing that mental health problems in adolescents with CPHC are associated with deteriorated self-perception.

Heterogeneous evidence has been found for parent–child relationships in children and adolescents with CPHC. While on the one hand, lower emotional warmth and higher levels of control and overprotection were found in families with a child that has a physical illness, higher levels of neglectful parenting and both higher and lower levels of authoritative parenting style were also observed compared to families with healthy children [55]. We found no differences with regards to parent relation between adolescents with and without a chronic condition, only in the subgroup with a double burden of mental problems and chronic illness. This finding adds to previous findings and may explain conflicting results. Parental responsiveness (warmth and support) plays a crucial role in psychological development in general and in the adaption to the physical illness specifically and seems to be impaired in the group with the double burden of suffering from a chronic illness and a mental health problem. High expressed emotion in interpersonal interactions is a well-known maladaptive behavior and a factor contributing to the maintenance of psychiatric disorders [56]. Hence, psychological support should also be provided for parents when needed. A supportive family environment providing support when needed and guaranteeing autonomy when possible in the period of adolescence should be a further aim in psychosocial support in this population. Psychosocial care requirements for children and adolescents are integrated in clinical practice guidelines for some chronic physical conditions such as type 1 diabetes [57], while others remain in development [58].

Moreover, peer relations, social acceptance (as opposed to bullying), and school environment/school performance are markedly deteriorated in the group with a double burden of mental health problems and a chronic illness. The Avon Longitudinal Study of Parents and Children exploring mediating factors of the co-occurrence of mental illness in children and adolescence suggests that a chronic illness may impact functioning and social development in early adolescence and consequently may lead to the development of mental illness. In particular, school absenteeism and peer victimization increase this risk over time [15]. Peer victimization and school disconnectedness have been associated with mental health problems rather than with CPHC [59]. Interventions fostering positive peer relationships and opportunities for social inclusion are necessary to avoid bullying and problems at school.

International surveys revealed a decreased HrQoL in children and adolescents with chronic conditions and mental health problems [60,61,62,63]. The German KIGGS study highlighted youths with neuro-dermatitis, obesity, and mental health problems as at-risk groups for deteriorated HrQoL and as target populations for prevention programs [42]. Both CPHC and mental conditions need to be targeted for treatment to reduce their possible joint disability burden [44].

For future research, a broad range of psychosocial risk factors for the comorbidity of chronic physical health conditions and mental health problems should be analyzed in prospective long-term cohort studies so that causal relations can be drawn.

This study has the following limitations. First, the response rate of 47.3% is low. To detect potential differences between participating and non-participating adolescents, we used teachers’ ratings for all adolescents [32]. Non-participating students showed slightly higher school absenteeism and more concentration problems, were less socially integrated and more socially withdrawn at school, and showed more behavioral problems. However, effect sizes were very low, and thus we expect a small response bias only. Second, the cross-sectional design does not allow causal conclusions for predictor analyses, and potential predictors have to be interpreted with caution. Rather, the findings from this study should be primarily interpreted in terms of correlations. Third, we exclusively used adolescent self-reports to assess CPHC and mental health problems. While the reliance on youth information is useful for internalizing problems, there are other problem areas where parent or teacher information might be more valid (i.e., such as externalizing problems, attention problems, or family SES). Thus, externalizing problems might be underreported in both populations. Moreover, whether mental health problems lead to deteriorated HrQoL aspects or deteriorated HrQol aspects to mental health problems cannot be answered. Furthermore, the validity and reliability of CPHC diagnoses can not be regarded as high as when assessed in a clinical interview. Due to the low number of cases for specific CPHC, we were not able to answer whether the risk for mental health problems is elevated in adolescents with specific CPHC.

## 5. Conclusions

The results from the present study have the following implications: First, there is a high need for identifying mental health problems in adolescents with CPHC, and therefore, screening instruments should be implemented in routine service care in pediatric clinics. This seems all the more important, as the COVID-19 crisis might have further increased mental health problems in this population. Second, a mental health screening in this population should not only include the broad assessment of symptoms of psychiatric disorders, but also the assessment of significant life events in the past and adverse psychosocial circumstances (e.g., physical or mental health disorders in other family members), as these factors seem to be substantially associated with mental health. Third, early interventions for those positively screened for mental health problems or with deteriorated quality of life should be provided in order to prevent further deterioration in well-being with all psychosocial consequences (educational and work performance). Hence, the availability of multidisciplinary care is important. A stepped-care approach for children and adolescents with chronic physical health conditions also seems important. Such an approach should span the whole spectrum from universal prevention for those with currently no mental health problems to selected prevention for those with well-known risk factors for mental health problems. Thus, those with mental health problems in the early stage could receive preventative treatment and those with more severe psychiatric symptoms could be referred to psychotherapeutic treatment. The use of online support services and e-health approaches might be particularly attractive for young people, ranging from appointment and medication reminders to delivering interventions such as online cognitive therapy and self-management strategies [2,64]. Indeed, the COVID-19 pandemic was also regarded as a catalyst for digital health interventions, and this included the population of adolescents with chronic conditions [65]. Fourth, apart from mental health problems, HrQoL should be a key target variable for evaluating the effectiveness of (preventive) interventions in this population. Implementing targeted risk-reduction strategies in clinical practice should not only reduce mental disorders in youths with a chronic physical health condition but also create optimal conditions for the best possible quality of life [64].

## Figures and Tables

**Table 1:** Prevalence of chronic physical health conditions in the total sample and by gender.

	Weighted Prevalence ^1^		
	Total (n = 3469)	Boys (n = 1544)	Girls (n = 1925)
Any chronic physical health conditions	287 (8.3%)	110 (7.1%)	182 (9.4%)
Diabetes/metabolic disease	10 (0.3%)	5 (0.3%)	5 (0.3%)
Hypertension	2 (0.1%)	2 (0.1%)	0 (0.0%)
Arthritis/rheumatism	11 (0.3%)	2 (0.1%)	9 (0.5%)
Paralysis	1 (<0.1%)	1 (0.1%)	0 (0.0%)
Asthma/bronchitis	65 (1.9%)	32 (2.1%)	32 (1.7%)
Epilepsy	5 (0.1%)	2 (0.2%)	2 (0.1%)
Migraine/headache	49 (1.4%)	14 (0.9%)	37 (1.9%)
Orthopedic disease	65 (1.9%)	21 (1.3%)	46 (2.4%)
Thyroid disease	21 (0.6%)	8 (0.5%)	12 (0.6%)
Cancer/tumor disease	3 (0.1%)	1 (0.1%)	3 (0.1%)
Heart disease	18 (0.5%)	7 (0.4%)	12 (0.6%)
Enteropathy	4 (0.1%)	0 (0.0%)	4 (0.2%)
Allergy ^2^	63 (1.8%)	27 (1.8%)	36 (1.9%)
Disability	18 (0.5%)	8 (0.5%)	10 (0.5%)
Other	33 (0.9%)	15 (1.0%)	18 (0.9%)

^1^ Weighted by gender and age group (inverse probability weighting). ^2^ Only counted if regular medical consultation or medication intake was necessary.

**Table 2:** Sociodemographic characteristics in adolescents with and without a chronic physical health condition.

	Adolescent without Chronic Physical Health Conditions (n = 3.170)	Adolescent with Chronic Physical Health Conditions (n = 311)	chi^2^ (df)	*p*
Gender			9.983 (1)	0.002
boys	1432 (45.3%)	112 (36.0%)		
girls	1726 (54.7%)	199 (64.0%)		
missing	12	0		
Age group			18.529 (3)	<0.001
5th grade	503 (15.9%)	28 (9.0%)		
7th grade	822 (25.9%)	66 (21.2%)		
9th grade	994 (31.4%)	110 (35.4%)		
11th grade	851 (26.8%)	107 (34.4%)		
Socioeconomic status (SES)			0.569 (2)	0.753
low SES	62 (2.0%)	6 (2.0%)		
medium SES	772 (25.0%)	71 (23.1%)		
high SES	2248 (72.9%)	230 (74.9%)		
missing	88	4		
Migration background ^1^			0.237 (1)	0.626
no	2307 (74.1%)	230 (75.4%)		
yes	805 (25.9%)	75 (24.6%)		
missing	58	6		
Residency ^2^			2.760 (1)	0.097
urban	1819 (58.2%)	193 (63.1%)		
rural	1308 (41.8%)	113 (36.9%)		
missing	43	5		
Family status			2.137 (2)	0.343
living with both parents	2293 (74.5%)	212 (70.9%)		
living with single parent	506 (16.4%)	54 (18.1%)		
living in patchwork family	277 (9.0%)	33 (11.0%)		
missing	94	12		
Parental employment			0.055 (2)	0.973
both parents	2442 (78.1%)	239 (78.1%)		
one parent	598 (19.1%)	58 (19.0%)		
no parent	85 (2.7%)	9 (2.9%)		
missing	45	5		
Chronic physical health conditions in a parent or sibling			224.665 (2)	<0.001
no	2397 (76.9%)	126 (41.4%)		
yes	392 (12.6%)	132 (43.4%)		
do not know	327 (10.5%)	46 (15.1%)		
missing	54	7		
Psychiatric disorder in a parent or sibling			22.470 (2)	<0.001
no	2753 (87.8%)	237 (78.2%)		
yes	125 (4.0%)	22 (7.3%)		
do not know	257 (8.2%)	44 (14.5%)		
missing	35	8		
Self-reported school performance			8.645 (3)	0.034
very good	682 (21.9%)	49 (16.1%)		
good	1300 (41.7%)	129 (42.3%)		
mediocre	1031 (33.1%)	120 (39.3%)		
below average	105 (3.4%)	7 (2.3%)		
missing	52	6		

^1^ Migration status if either the adolescent or one parent was not born in Austria. ^2^ Urban: living in a region with ≥ 10,000 inhabitants; rural: living in a region with < 10,000 inhabitants.

**Table 3:** Multivariate logistic regression model predicting clinically relevant mental health problems in adolescents with a chronic physical health condition.

Predictor	*b* (SE)	Wald	*p*	Odds Ratio (OR)	OR 95% CI
Constant		5.718	0.057		
Psychiatric disorder of parent or sibling (Ref. no)					
do not know	0.587 (0.36)	2.735	0.098	1.80	[0.90; 3.61]
yes	0.995 (0.52)	3.677	0.055	2.71	[0.98; 7.48]
Any potentially traumatizing life event (Ref. no)	1.212 (0.39)	9.914	**0.002**	3.36	[1.58; 7.15]
More than one chronic physical health condition (Ref. Single chronic physical health condition)	0.018 (0.39)	0.003	0.956	1.02	[0.54; 1.92]
Needs regular medication intake due to chronic disease (Ref. no)	0.926 (0.29)	10.570	**0.001**	2.52	[1.44; 1.41]

Note: Numbers in bold indicate statistically significant *p*-values (<0.05).

**Table 4:** Health-related quality of life scores in adolescents with a chronic physical health condition with and without comorbid mental health problems compared to a healthy control group.

	Mean (SD)			Test Statistic		
	Healthy Adolescents ^1^ A	Adolescents with a Chronic Physical Health Condition without Comorbid Mental Health Problems ^2^ B	Adolescents a Chronic Physical Health Condition and Comorbid Mental Health Problems ^3^ C	*F*	*p*	Tukey Post-Hoc-Tests
KIDSCREEN-10	54.34 (8.93)	51.44 (8.82)	39.23 (10.85)	156.495	<0.001	A,B > C; A > B
Self-Perception	51.51 (8.55)	49.88 (9.16)	40.70 (9.80)	88.947	<0.001	A,B > C; A > B
Parent Relation and Home Life	54.76 (7.94)	53.35 (8.00)	44.22 (11.68)	94.615	<0.001	A,B > C
Social Support and Peers	53.66 (8.11)	52.38 (9.27)	47.52 (10.63)	31.798	<0.001	A,B > C
School Environment	53.58 (8.66)	51.61 (8.24)	44.39 (9.53)	64.395	<0.001	A,B > C; A > B
Bullying (Social Acceptance)	52.74 (7.71)	53.05 (7.12)	45.13 (14.06)	50.143	<0.001	A,B > C

^1^ Depending on the data availability, the sample size varies between 2172 and 2286. ^2^ Depending on the data availability, the sample size varies between 175 and 181. ^3^ Depending on the data availability, the sample size varies between 114 and 118.

## Data Availability

The data that support the findings of this study are available from the corresponding author upon reasonable request.

## Supplementary Materials

The following supporting information can be downloaded at https://www.mdpi.com/article/10.3390/jcm12051927/s1, Table S1: Prevalence of mental health risk in adolescents with and without chronic diseases; Table S2: Univariate logistic regression predicting clinically relevant mental health problems in adolescents with a chronic disease.

## References

[1] Michaud P.A., Suris J.C., Viner R. (2007). The Adolescent with a Chronic Condition: Epidemiology, Developmental Issues and Health Care Provision.

[2] Sawyer S.M., Drew S., Yeo M.S., Britto M.T. (2007). Adolescents with a chronic condition: Challenges living, challenges treating. Lancet.

[3] Silva N., Pereira M., Otto C., Ravens-Sieberer U., Canavarro M.C., Bullinger M. (2019). Do 8- to 18-year-old children/adolescents with chronic physical health conditions have worse health-related quality of life than their healthy peers? a meta-analysis of studies using the KIDSCREEN questionnaires. Qual. Life Res..

[4] Laurens K.R., Green M.J., Dean K., Tzoumakis S., Harris F., Islam F., Kariuki M., Essery C.M., Schofield J.M., Carr V.J. (2019). Chronic Physical Health Conditions, Mental Health, and Sources of Support in a Longitudinal Australian Child Population Cohort. J. Pediatr. Psychol..

[5] Felder-Puig R., Teutsch F., Ramelow D., Maier G. (2018). Gesundheit Und Gesundheitsverhalten von Österreichischen Schülerinnen Und Schülern: Ergebnisse Des WHO-HBSC-Survey 2018. https://www.sozialministerium.at/dam/jcr:0f4973f8-dc8b-4227-9e64-c76cec64b343/2018%20HBSC-Bericht%20mit%20Alternativtexten_final.pdf.

[6] Ramelow D., Felder-Puig R. (2013). HBSC Factsheet Nr. 07/2013: Die Psychische Gesundheit von Österreichischen Schülerinnen und Schülern: Ergebnisse 2010 und Trends. https://www.google.com/url?sa=t&rct=j&q=&esrc=s&source=web&cd=&ved=2ahUKEwj50uCK4_D5AhVlXfEDHaMtBngQFnoECA-YQAQ&url=http%3A%2F%2Fwww.wiengs.at%2Ffileadmin%2Fuser_upload%2FFactsheets%2FLBIHPR__2013__Factsheet_Nr7_psychische_Gesundheit.pdf&usg=AOvVaw2Z4gvXv7R89xT8yyNxHnQC.

[7] Dür W., Griebler R. (2007). Die Gesundheit Der Österreichischen SchülerInnen Im Lebenszusammenhang: Ergebnisse Des WHO-HBSC-Survey 2006. https://www.sozialministerium.at/dam/jcr:e5e56b2c-b7d5-4f74-99ba-2af4b4101991/bericht_hbsc_2007_gesamt_mit_anhang907.pdf.

[8] Newacheck P.W., Taylor W.R. (1992). Childhood chronic illness: Prevalence, severity, and impact. Am. J. Public Health.

[9] Nibras S., Kentor R., Masood Y., Price K., Schneider N.M., Tenenbaum R.B., Calarge C. (2022). Psychological and Psychiatric Comorbidities in Youth with Serious Physical Illness. Children.

[10] Vessey J.A. (1999). Psychological comorbidity in children with chronic conditions. Pediatr. Nurs..

[11] (1993). Committee on Children with Disabilities and Committee on Psychosocial Aspects of Child and Family Health Psychosocial risks of chronic health conditions in childhood and adolescence. Pediatrics.

[12] Delamater A.M., Guzman A., Aparicio K. (2017). Mental health issues in children and adolescents with chronic illness. Int. J. Hum. Rights Healthc..

[13] Jones L.C., Mrug S., Elliott M.N., Toomey S.L., Tortolero S., Schuster M.A. (2017). Chronic Physical Health Conditions and Emotional Problems From Early Adolescence Through Midadolescence. Acad. Pediatr..

[14] Brady A.M., Deighton J., Stansfeld S. (2017). Psychiatric outcomes associated with chronic illness in adolescence: A systematic review. J. Adolesc..

[15] Brady A.M., Deighton J., Stansfeld S. (2021). Chronic illness in childhood and early adolescence: A longitudinal exploration of co-occurring mental illness. Dev. Psychopathol..

[16] Määttä H., Honkanen M., Hurtig T., Taanila A., Ebeling H., Koivumaa-Honkanen H. (2022). Childhood chronic condition and subsequent self-reported internalizing and externalizing problems in adolescence: A birth cohort study. Eur. J. Pediatr..

[17] Pinquart M., Shen Y. (2011). Behavior Problems in Children and Adolescents With Chronic Physical Illness: A Meta-Analysis. J. Pediatr. Psychol..

[18] Schmidt S., Petersen C., Bullinger M. (2003). Coping with chronic disease from the perspective of children and adolescents–A conceptual framework and its implications for participation: Coping with chronic disease. Child Care Health Dev..

[19] Stein R.E., Jessop D.J. (1982). A noncategorical approach to chronic childhood illness. Public Health Rep..

[20] Stam H., Hartman E.E., Deurloo J.A., Groothoff J., Grootenhuis M.A. (2006). Young Adult Patients with a History of Pediatric Disease: Impact on Course of Life and Transition into Adulthood. J. Adolesc. Health.

[21] Taylor R.M., Gibson F., Franck L.S. (2008). The experience of living with a chronic illness during adolescence: A critical review of the literature. J. Clin. Nurs..

[22] Sentenac M., Arnaud C., Gavin A., Molcho M., Gabhainn S.N., Godeau E. (2012). Peer Victimization Among School-aged Children With Chronic Conditions. Epidemiol. Rev..

[23] Reinauer C., Viermann R., Förtsch K., Linderskamp H., Warschburger P., Holl R.W., Staab D., Minden K., Muche R., COACH Consortium (2018). Motivational Interviewing as a tool to enhance access to mental health treatment in adolescents with chronic medical conditions and need for psychological support (COACH-MI): Study protocol for a clusterrandomised controlled trial. Trials.

[24] Reinauer C., Platzbecker A.L., Viermann R., Domhardt M., Baumeister H., Foertsch K., Linderskamp H., Krassuski L., Staab D., Minden K. (2021). Efficacy of Motivational Interviewing to Improve Utilization of Mental Health Services Among Youths With Chronic Medical Conditions. JAMA Netw. Open.

[25] Ravens-Sieberer U., Gosch A., Abel T., Auquier P., Bellach B.-M., Bruil J., Dür W., Power M., Rajmil L. (2001). The European KIDSCREEN Group Quality of life in children and adolescents: A European public health perspective. Soz.-Präventivmedizin.

[26] Gaspar T., Ribeiro J.P., de Matos M.G., Leal I., Ferreira A. (2012). Health-Related Quality of Life in Children and Adolescents: Subjective Well Being. Span. J. Psychol..

[27] Berkelbach van der Sprenkel E.E., Nijhof S.L., Dalmeijer G.W., Onland-Moret N.C., de Roos S.A., Lesscher H.M.B., van de Putte E.M., van der Ent C.K., Finkenauer C., Stevens G.W.J.M. (2022). Psychosocial functioning in adolescents growing up with chronic disease: The Dutch HBSC study. Eur. J. Pediatr..

[28] Wagner G., Berger G., Sinnreich U., Grylli V., Schober E., Huber W.-D., Karwautz A. (2008). Quality of Life in Adolescents With Treated Coeliac Disease: Influence of Compliance and Age at Diagnosis. J. Pediatr. Gastroenterol. Nutr..

[29] Zeiler M., Peer S., Philipp J., Truttmann S., Wagner G., Karwautz A., Waldherr K. (2021). Web-Based Versus Paper-Pencil Assessment of Behavioral Problems Using the Youth Self-Report. Eur. J. Psychol. Assess..

[30] Philipp J., Zeiler M., Waldherr K., Nitsch M., Dür W., Karwautz A., Wagner G. (2014). The Mental Health in Austrian Teenagers (MHAT)-Study: Preliminary results from a pilot study. Neuropsychiatrie.

[31] Zeiler M., Wagner G., Philipp J., Nitsch M., Truttmann S., Dür W., Karwautz A., Waldherr K. (2018). The Mental Health in Austrian Teenagers (MHAT) Study: Design, methodology, description of study population. Neuropsychiatrie.

[32] Wagner G., Zeiler M., Waldherr K., Philipp J., Truttmann S., Dür W., Treasure J.L., Karwautz A.F.K. (2017). Mental health problems in Austrian adolescents: A nationwide, two-stage epidemiological study applying DSM-5 criteria. Eur. Child Adolesc. Psychiatry.

[33] Boyce W., Torsheim T., Currie C., Zambon A. (2006). The Family Affluence Scale as a Measure of National Wealth: Validation of an Adolescent Self-Report Measure. Soc. Indic. Res..

[34] Achenbach T.M. (1991). Manual for the Youth Self-Report and 1991 Profile.

[35] von M. Döpfner B., Plück J., Bölte S., Lenz K., Melchers P. (1991). Arbeitsgruppe Deutsche Child Behavior Checklist Fragebogen für Jugendliche. Deutsche Bearbeitung der Youth Self-Report Form der Child Behavior Checklist (YSR). Einführung und Anleitung zur Handauswertung mit deutschen Normen.

[36] Rescorla L., Ivanova M.Y., Achenbach T.M., Begovac I., Chahed M., Drugli M.B., Emerich D.R., Fung D.S.S., Haider M., Hansson K. (2012). International Epidemiology of Child and Adolescent Psychopathology II: Integration and Applications of Dimensional Findings From 44 Societies. J. Am. Acad. Child Adolesc. Psychiatry.

[37] Ravens-Sieberer U., Gosch A., Rajmil L., Erhart M., Bruil J., Power M., Duer W., Auquier P., Cloetta B., Czemy L. (2008). The KIDSCREEN-52 quality of life measure for children and adolescents: Psychometric results from a cross-cultural survey in 13 European countries. Value Health.

[38] Cobham V.E., Hickling A., Kimball H., Thomas H.J., Scott J.G., Middeldorp C.M. (2020). Systematic Review: Anxiety in Children and Adolescents With Chronic Medical Conditions. J. Am. Acad. Child Adolesc. Psychiatry.

[39] Tegethoff M., Belardi A., Stalujanis E., Meinlschmidt G. (2015). Association between mental disorders and physical diseases in adolescents from a nationally representative cohort. Psychosom. Med..

[40] Berger G., Waldhoer T., Barrientos I., Kunkel D., Rami-Merhar B.M., Schober E., Karwautz A., Wagner G. (2019). Association of insulin-manipulation and psychiatric disorders: A systematic epidemiological evaluation of adolescents with type 1 diabetes in Austria. Pediatr. Diabetes.

[41] Kline-Simon A.H., Weisner C., Sterling S. (2016). Point Prevalence of Co-Occurring Behavioral Health Conditions and Associated Chronic Disease Burden Among Adolescents. J. Am. Acad. Child Adolesc. Psychiatry.

[42] Zheng K., Abraham C., Bruzzese J.-M., Smaldone A. (2020). Longitudinal Relationships Between Depression and Chronic Illness in Adolescents: An Integrative Review. J. Pediatr. Health Care.

[43] Stein M.B., Cox B.J., Afifi T.O., Belik S.-L., Sareen J. (2006). Does co-morbid depressive illness magnify the impact of chronic physical illness? A population-based perspective. Psychol. Med..

[44] Scott K.M., Von Korff M., Alonso J., Angermeyer M.C., Bromet E., Fayyad J., de Girolamo G., Demyttenaere K., Gasquet I., Gureje O. (2009). Mental–physical co-morbidity and its relationship with disability: Results from the World Mental Health Surveys. Psychol. Med..

[45] Jones R., Hiscock H., Wurzel D., Kao K.-T., Freeman J.L., Ride J. (2022). Mental healthcare for children with chronic conditions: A qualitative study. Arch. Dis. Child..

[46] Lunkenheimer F., Domhardt M., Geirhos A., Kilian R., Mueller-Stierlin A.S., Holl R.W., Meissner T., Minden K., Moshagen M., Ranz R. (2020). Effectiveness and cost-effectiveness of guided Internet- and mobile-based CBT for adolescents and young adults with chronic somatic conditions and comorbid depression and anxiety symptoms (youthCOACHCD): Study protocol for a multicentre randomized controlled trial. Trials.

[47] Zeiler M., Wittek T., Graf T., Bozic I., Nitsch M., Waldherr K., Karwautz A., Wagner G., Berger G. (2022). Psychosocial impact of the COVID-19 pandemic for adolescents with type-1-diabetes: A qualitative interview study involving adolescents and parents. Behav. Med..

[48] Jones B., Woolfenden S., Pengilly S., Breen C., Cohn R., Biviano L., Johns A., Worth A., Lamb R., Lingam R. (2020). COVID-19 pandemic: The impact on vulnerable children and young people in Australia. J. Paediatr. Child Health.

[49] Cay M., Gonzalez-Heydrich J., Teicher M.H., van der Heijden H., Ongur D., Shinn A.K., Upadhyay J. (2022). Childhood maltreatment and its role in the development of pain and psychopathology. Lancet Child Adolesc. Health.

[50] Overstreet C., Berenz E.C., Kendler K.S., Dick D.M., Amstadter A.B. (2017). Predictors and mental health outcomes of potentially traumatic event exposure. Psychiatry Res..

[51] Kessler R.C., Avenevoli S., Costello E.J., Georgiades K., Green J.G., Gruber M.J., He J., Koretz D., McLaughlin K.A., Petukhova M. (2012). Prevalence, Persistence, and Sociodemographic Correlates of DSM-IV Disorders in the National Comorbidity Survey Replication Adolescent Supplement. Arch. Gen. Psychiatry.

[52] Yung A.R. (2016). Youth services: The need to integrate mental health, physical health and social care: Commentary on Malla et al.: From early intervention in psychosis to youth mental health reform: A review of the evolution and transformation of mental health services for young people. Soc. Psychiatry Psychiatr. Epidemiol..

[53] Barlow J.H., Ellard D.R. (2006). The psychosocial well-being of children with chronic disease, their parents and siblings: An overview of the research evidence base. Child Care Health Dev..

[54] Pinquart M. (2013). Self-esteem of children and adolescents with chronic illness: A meta-analysis: Self-esteem and chronic illness. Child Care Health Dev..

[55] Pinquart M. (2013). Do the Parent–Child Relationship and Parenting Behaviors Differ Between Families With a Child With and Without Chronic Illness? A Meta-Analysis. J. Pediatr. Psychol..

[56] Philipp J., Truttmann S., Zeiler M., Franta C., Wittek T., Schöfbeck G., Mitterer M., Mairhofer D., Zanko A., Imgart H. (2020). Reduction of High Expressed Emotion and Treatment Outcomes in Anorexia Nervosa-Caregivers’ and Adolescents’ Perspective. J. Clin. Med..

[57] Delamater A.M., de Wit M., McDarby V., Malik J.A., Hilliard M.E., Northam E., Acerini C.L. (2018). ISPAD Clinical Practice Consensus Guidelines 2018: Psychological care of children and adolescents with type 1 diabetes. Pediatr. Diabetes.

[58] Coburn S., Rose M., Streisand R., Sady M., Parker M., Suslovic W., Weisbrod V., Kerzner B., Kahn I. (2020). Psychological Needs and Services in a Pediatric Multidisciplinary Celiac Disease Clinic. J. Clin. Psychol. Med. Settings.

[59] James C., Corman H., Noonan K., Reichman N.E., Jimenez M.E. (2022). Adolescent Chronic Health Conditions and School Disconnectedness. J. Dev. Behav. Pediatr. JDBP.

[60] Bai G., Herten M.H., Landgraf J.M., Korfage I.J., Raat H. (2017). Childhood chronic conditions and health-related quality of life: Findings from a large population-based study. PLoS ONE.

[61] Hölling H., Schlack R., Dippelhofer A., Kurth B.-M. (2008). Personale, familiäre und soziale Schutzfaktoren und gesundheitsbezogene Lebensqualität chronisch kranker Kinder und Jugendlicher. Bundesgesundheitsblatt-Gesundh.-Gesundh..

[62] Varni J.W., Limbers C.A., Burwinkle T.M. (2007). Impaired health-related quality of life in children and adolescents with chronic conditions: A comparative analysis of 10 disease clusters and 33 disease categories/severities utilizing the PedsQL^TM^ 4.0 Generic Core Scales. Health Qual. Life Outcomes.

[63] Ravens-Sieberer U., Erhart M., Wille N., Bullinger M., The BELLA Study Group (2008). Health-related quality of life in children and adolescents in Germany: Results of the BELLA study. Eur. Child Adolesc. Psychiatry.

[64] Ferro M.A., Lipman E.L., Van Lieshout R.J., Gorter J.W., Shanahan L., Boyle M., Georgiades K., Timmons B. (2019). Multimorbidity in Children and Youth Across the Life-course (MY LIFE): Protocol of a Canadian prospective study. BMJ Open.

[65] Danne T., Limbert C. (2020). COVID-19, type 1 diabetes, and technology: Why paediatric patients are leading the way. Lancet Diabetes Endocrinol..

