# Supplementary material for: Mental Health and Health-Related Quality of Life in Austrian Adolescents with Chronic Physical Health Conditions: Results from the MHAT Study
Source: J Clin Med. 2023 Feb 28;12(5):1927. doi: 10.3390/jcm12051927 (PMC10003709; doi:10.3390/jcm12051927)
Supplement: Supplementary file 1 [file jcm-12-01927-s001.zip › jcm-2200886-supplementary.pdf]

Supplement Table S1. Prevalence of mental health risk in adolescents with and without a chronic physical health condition

	Mean[a] (SD)		Test statistic		Effect size	
	**Adolescent without chronic physical health conditions (n = 3087)**	**Adolescent with chronic physical health conditions (n = 303)**	***t* (df=3388)**	***p***	**Cohen's *d***	**95% CI**
YSR Total	54.46 (8.65)	58.92 (8.67)	8.573	< .001	0.52	[0.40; 0.64]
Internalizing	54.52 (9.65)	59.86 (9.50)	9.216	< .001	0.56	[0.44; 0.67]
Externalizing	51.87 (8.03)	54.47 (8.16)	5.366	< .001	0.32	[0.21; 0.44]
Social withdrawn	55.66 (7.05)	57.75 (7.78)	4.886	< .001	0.29	[0.18; 0.41]
Somatic complaints	57.74 (7.33)	62.20 (8.43)	9.967	< .001	0.60	[0.48; 0.72]
Anxious/depressed	55.41 (7.10)	58.77 (8.26)	7.747	< .001	0.47	[0.35; 0.59]
Social problems	54.96 (6.32)	56.36 (7.44)	3.629	< .001	0.22	[0.10; 0.34]
Thought problems	56.89 (7.85)	59.41 (8.33)	5.304	< .001	0.32	[0.20; 0.44]
Attention problems	56.00 (6.71)	57.77 (7.28)	4.328	< .001	0.26	[0.14; 0.38]
Dissocial behavior	54.59 (5.93)	56.61 (6.84)	5.587	< .001	0.34	[0.22; 0.46]
Aggressive behavior	54.07 (5.41)	55.44 (5.89)	4.150	< .001	0.25	[0.13; 0.37]

[a] T-values

Supplement Table S2. Univariate logistic regression predicting clinically relevant mental health problems in adolescents with a chronic physical health condition (slopes presented only)

Predictor	*b (SE)*	Wald	*p*	OR	OR 95% CI
Females (Ref. Male)	0.359 (0.25)	2.104	.147	1.43	[0.88; 2.33]
Age group (Ref. = 5th grade)					
7th grade	-0.360 (0.48)	0.573	.449	0.70	[0.28; 1.77]
9th grade	-0.360 (0.45)	0.184	.668	0.83	[0.35; 1.98]
11th grade	0.083 (0.44)	0.035	.852	1.09	[0.46; 2.59]
Family status (Ref. = living with both parents)					
living with single parent	0.488 (0.31)	2.435	.119	1.63	[0.88; 3.01]
living in patchwork family	0.314 (0.39)	0.667	.414	1.37	[0.64; 2.91]
Migration (Ref. No)	0.244 (0.27)	0.798	.372	1.28	[0.75; 2.18]
Low or medium socioeconomic status (Ref. = high)	-0.469 (0.27)	0.364	.546	1.18	[0.69; 2.02]
Urban place of residence (Ref. Rural)	0.058 (0.25)	0.057	.812	1.06	[0.66; 1.72]
No or one parent employed (Ref. Both parents)	0.436 (0.29)	2.321	.128	1.55	[0.88; 2.71]
Chronic somatic disease of parent or sibling (Ref. no)					
do not know	0.214 (0.35)	0.366	.545	1.24	[0.62; 2.48]
yes	0.213 (0.26)	0.681	.409	1.24	[0.75; 2.05]
Psychiatric disorder of parent or sibling (Ref. no)					
do not know	0.529 (0.33)	2.561	.110	1.70	[0.89; 3.25]
yes	0.935 (0.48)	3.852	**.050**	2.55	[1.00; 6.48]
Any burdensome lifetime event (Ref. No)	-0.087 (0.24)	0.135	.713	0.92	[0.58; 1.46]
Any traumatic lifetime event (ref. No)	1.186 (0.36)	11.104	**.001**	3.28	[1.63; 6.58]
Age of chronic disease onset	-0.001 (0.023)	0.001	.972	1.00	[0.96; 1.05]
More than one somatic chronic disease (Ref. Single chronic disease)	0.472 (0.28)	2.894	.089	1.60	[0.93; 2.76]
Needs regular medical checks due to chronic disease (Ref. No)	0.161 (0.25)	0.426	.514	1.18	[0.72; 1.91]
Needs regular medication intake due to chronic disease (Ref. No)	0.833 (0.26)	10.600	**.001**	2.30	[1.39; 3.80]

Note: Numbers in bold indicate statistically significant *p*-values (≤ 0.05).
